# Metabolomics and Communication Skills Development in Children; Evidence from the Ages and Stages Questionnaire

**DOI:** 10.3390/metabo9030042

**Published:** 2019-03-05

**Authors:** Rachel S. Kelly, Adrianna Boulin, Nancy Laranjo, Kathleen Lee-Sarwar, Su H. Chu, Aishwarya P. Yadama, Vincent Carey, Augusto A. Litonjua, Jessica Lasky-Su, Scott T. Weiss

**Affiliations:** 1 Channing Division of Network Medicine, Department of Medicine, Brigham and Women’s Hospital and Harvard Medical School, Boston, MA 02115, USA; hprke@channing.harvard.edu (R.S.K.); aboulin@fenwayhealth.org (A.B.); renml@channing.harvard.edu (N.L.); klee-sarwar@bwh.harvard.edu (K.L.-S.); rechu@channing.harvard.edu (S.H.C.); nhaya@channing.harvard.edu (A.P.Y.); stvjc@channing.harvard.edu (V.C.); rejas@channing.harvard.edu (J.L.-S.); 2 The Fenway Institute, Fenway Health, Boston, MA 02215, USA; 3 Division of Rheumatology, Immunology and Allergy, Brigham and Women’s Hospital, Boston, MA 02115, USA; 4 Division of Pediatric Pulmonary Medicine, Golisano Children’s Hospital at Strong, University of Rochester Medical Center, Rochester, NY 14642, USA; Augusto_Litonjua@urmc.rochester.edu

**Keywords:** metabolomics, ages and stages questionnaire (ASQ), autism, endocannabinoid, serotonin, tryptophan metabolism, tyrosine metabolism, N-formylanthranilic acid, childhood development

## Abstract

We hypothesized metabolomic profiling could be utilized to identify children who scored poorly on the communication component of the Ages and Stages Questionnaire (ASQ); which assesses development in childhood, and to provide candidate biomarkers for autism spectrum disorders (ASD). In a population of three-year-old children, 15 plasma metabolites, were significantly (*p* < 0.05) different between children who were categorized as having communication skills that were “on schedule” (*n* = 365 (90.6%)) as compared to those “requiring further monitoring/evaluation” (*n* = 38 (9.4%)) according to multivariable regression models. Five of these metabolites, including three endocannabinoids, were also dysregulated at age one (*n* = 204 “on schedule”, *n* = 24 “further monitoring/evaluation”) in the same children. Stool metabolomic profiling identified 11 significant metabolites. Both the plasma and stool results implicated a role for tryptophan and tyrosine metabolism; in particular, higher levels of N-formylanthranilic acid were associated with an improved communication score in both biosample types. A model based on the significant plasma metabolites demonstrated high sensitivity (88.9%) and specificity (84.5%) for the prediction of autism by age 8. These results provide evidence that ASQ communication score and metabolomic profiling of plasma and/or stool may provide alternative approaches for early diagnosis of ASD, as well as insights into the pathobiology of these conditions.

## 1. Introduction

Autism spectrum disorders (ASD) are a collection of heterogeneous neurodevelopmental disorders characterized by persistent deficits in social communication and interaction across multiple contexts, including social reciprocity, nonverbal communicative behaviors, and skills in developing, maintaining, and understanding relationships [1]. The Centers for Disease Control and Prevention recently released their bi-annual update of the prevalence of autism in the United States of America. With an estimated 15% increase in cases since 2016, they predicted the prevalence would be 1 in every 59 children in 2018 [2]. Current diagnostics involve behavioral observation and assessments of speech, language, and intellectual abilities. The average age of diagnosis in the United States is four years [2], although parents often report signs of developmental delay as early as 18 months [3]. Earlier detection could allow for more effective treatment in improving social, communicative, adaptive and cognitive outcomes [4].

A common assessment used to measure a child’s development between birth and the age of six is the Ages and Stages Questionnaire (ASQ). This tool assesses five developmental skills domains: communication, gross motor, fine motor, problem solving, and personal-social [5]. Evidence suggests that the ASQ can provide an effective screening tool to identify children at high risk of ASD. Specifically, it has been reported that utilizing a binary cut-off based on ASQ Communication score can identify 95% of at-risk children, as defined by the Modified Checklist for Autism in Toddlers (M-CHAT) [6]. Furthermore, it has been shown that the ASQ is less sensitive to demographic factors than other commonly used development assessment approaches [7].

Nevertheless, in addition to standardized screening, there is a need for a more sensitive means of ASD detection [8]. A growing body of research demonstrates differing metabolic patterns in ASD patients as compared to controls, in particular dysregulated levels of fatty acids, sterols, intermediary metabolites, phospholipids, and molecules associated with oxidative stress [9]. Similarly, an increased level of blood serotonin has been consistently linked with autism [10]. The development of metabolomics now offers the opportunity to characterize efficiently levels of a broad range of metabolites in a large number of subjects. Taken together, this suggests the potential for the development of metabolomic based biomarkers of ASD that could help close the gap in the identification and/or prediction of these disorders.

In this study, we investigate blood and stool metabolomic profiles associated with the ASQ derived communication score, as a proxy for ASD risk, in children from the Vitamin D Antenatal Asthma Reduction Trial (VDAART, clinicaltrials.gov Identifier: NCT00920621), a clinical trial of prenatal vitamin D supplementation and outcomes in pregnant women and their offspring [11]. We hypothesize that the metabolome of children with poor communication scores differs from that of children exhibiting “normal” communication scores, and that these differences involve biological pathways that are implicated in ASD.

## 2. Results

### 2.1. Study Population

In total, 403 children from VDAART had metabolomic profiling of plasma and an ASQ communication score at age three. The ASQ-3 communication score is based on six questions administered to parents regarding their child’s ability. Standardized scores are then compared to the expected mean score obtained from a reference distribution of scores, and the children are categorized as: (i) “On Schedule for developing normally” (above the mean); (ii) “Requires Monitoring” (1–2 standard deviations below the mean); (iii) “Needs further evaluation” (>2 standard deviations below the mean). For these analyses we focused on communication skill using a binary cut off comparing those “On schedule for developing normally” versus those either requiring “further evaluation” or “monitoring”, based on the recommendations of Hardy et al. [6]. The majority (*n* = 365, 90.6%) of VDAART children were categorized as “on schedule for developing normally” based on their ASQ communication score (Table 1). Of the 403 children, 215 also had plasma metabolomic profiling at age 1 (53.3%), and 228 (56.6%) had metabolomic profiling on a stool sample collected at age three (Supplementary Table S1).

Children in the lowest communication score category (“Requires further evaluation”) were significantly less likely to be from the San Diego VDAART site than from the Boston or St. Louis sites (*p* = 0.002), and there were borderline significant associations between score category with gender (*p* = 0.076) and with maternal educational level (*p* = 0.057). There was no significant association with either prenatal vitamin D supplementation or asthma/wheeze which were the primary exposure and endpoint of interest in the original VDAART study. Communication score was also assessed at the age one and age two visits, and while there appeared to be a strong relationship between communication category when considering the age three and the age two visit (*p* = 8.0 × 10^−9^), this was not apparent with the age one visit (Table 1; Supplementary Figure S1). In addition to communication skills, the ASQ also assess four other domains using the same six question approach; gross motor skills, fine motor skills, personal social skills and problem solving skills. Communication score at age three was significantly correlated with the scores in the other four ASQ assessed categories, with the strongest relationships for personal-social skill score (*r* = 0.45, *p* < 2.2 × 10^−16^) and problem solving score (*r* = 0.46, *p ≤* 2.2 × 10^−16^) (Supplementary Table S2).

### 2.2. Plasma Metabolome

After QC and data processing, 481 metabolites were retained for analysis in the age three-year plasma samples (Supplementary Table S3). Partial Least Squares Discriminant analysis (PLS-DA) revealed no evidence of a global difference in the metabolomes of children who were “on schedule for developing normally” versus those in the “Monitoring” and “further evaluation groups”; and therefore no discriminatory ability. The PLS-DA scores plot of the first two components shows no obvious separation between the two groups (Supplementary Figure S2), and this is confirmed by the PLS-DA metrics; R^2^ = 0.13; Q^2^ = −0.21; permutation *p*-value = 0.994. However, multivariable logistic regression models, adjusting for a child’s race, child’s sex, maternal education, maternal marital status, treatment group and clinical site, identified 15 significant metabolites. Five metabolites were significantly lower in children in the combined lowest two categories of communication score (requiring further monitoring/evaluation), as compared to children on schedule for normal development. While 10 metabolites presented at significantly higher levels in the children in the lowest categories of communication score (Table 2). Interestingly, although there was no overall discriminatory ability when considering the whole metabolome; seven of the metabolites with the highest Variable Importance in the Projection (VIP) Scores from the PLS-DA (>2) were also among those identified as significant in the regression analyses including two metabolites of tryptophan metabolism and an endocannabinoid (Supplementary Figure S2). Sample size was limited for the three communication score categories model, nevertheless we also ran these analyses using three score categories as a continuous variable in a linear model and found that the majority (*n* = 9) of these metabolites retained significance. We used a “number of effective tests” approach [12,13] to account for multiple testing. Based on the 481 metabolites, a total of 12 principal components were needed to explain 50% of the variance in the data. Using a multiple testing corrected *p*-value threshold of 0.004 accounting for 12 effective tests, only N-formylphenylalanine, a metabolite of tyrosine metabolism, retained significance.

Given the differences in the prevalence of communication score category across the three study sites (Table 1), we also ran stratified analyses to determine the consistency of the metabolite-score associations by site. Analyses were unadjusted due to sample size constraints and resulting convergence issues and few reached significance, however the majority of associations were in the same direction of effect across the three cohorts, providing further support for the role of these metabolites (Supplemental Table S3). Only cinnamoylglycine and N-formylanthranilic acid were in a discordant direction of effect in San Diego as compared to Boston and St Louis, while prolylhydroxyproline was discordant in terms of direction of effect in the Boston children.

Of the 15 nominally significant metabolites, three were involved in tryptophan metabolism, three in endocannabinoid metabolism, and three were xenobiotics. The remaining metabolites were involved in tyrosine, fatty acid, phospholipid, sphingolipid, methionine, cysteine, SAM and taurine metabolism, and the urea cycle. Five of the 15 metabolites, oleoyl ethanolamide, palmitoyl ethanolamide, linoleoyl ethanolamide, sphingomyelin (d18:1/25:0, d19:0/24:1, d20:1/23:0, d19:1/24:0) and docosahexaenoylcarnitine (C22:6), were also significantly associated with age 3 binary communication score when measured in the age one samples from the 215 children with both age one and age three samples. All were lipids involved in endocannabinoid, sphingolipid and fatty acid metabolism. There were weak correlations between the measures of these metabolites at age 1 and age 3 (*rho* = 0.13 to 0.37, *p* = 2.4 × 10^−8^ to 0.064, Supplemental Table S4).

### 2.3. Stool Metabolome

In the 228 children who also had stool metabolomic profiling at age three, a total of 737 metabolites were quantified and passed QC (Supplementary Table S5), including 321 (66.7%) of the 481 metabolites that were measurable and passed QC in plasma. The distribution of communication scores in the subset of children with stool was comparable to the full plasma population; 89.5% (*n* = 204) of children were “on schedule for developing normally”. There were no significant differences in any of the explored baseline variables in the stool subset (Supplementary Table S1), although, as with the full population, there was a significant association between age two communication score category and age three communication score category (*p* = 2.3 × 10^−4^). Again, PLS-DA models failed to identify global metabolomic signatures with the ability to distinguish between children on schedule to develop normally from those requiring further monitoring or evaluation (R^2^ = 0.10, Q^2^ = −1.13, permuted *p*-value = 0.985) (Supplementary Figure S3).

In this subset of children, 13 metabolites were significantly associated with binary communication score; all were at lower levels in the children in the lowest two categories (Table 3). Again, these significant metabolites represented a number of tryptophan and tyrosine metabolites, including N-formylanthranilic acid, which was also identified as significant in the plasma, and two other metabolites of the Tryptophan metabolism pathway; N-acetylserotonin and 2-aminophenol. Of the 13 metabolites, seven had a VIP score greater than 2 in the PLS-DA analysis (Supplementary Figure S3) and all 13 had a score >1, indicating that both methods identified these metabolites as differing between children on schedule versus those requiring further monitoring and evaluation.

A total of 16 principal components were required to explain 50% of the variance in the 737 stool metabolites. Using a corrected *p*-value threshold of 0.003, only salicylate, which is synthetically produced for use in drugs such as aspirin, but which can also naturally occur in certain foods, particularly berry fruits and dried fruits, retained significance in the logistic regression model.

### 2.4. Correlation between the Plasma and Stool Metabolomic Results

**Figure 1:**
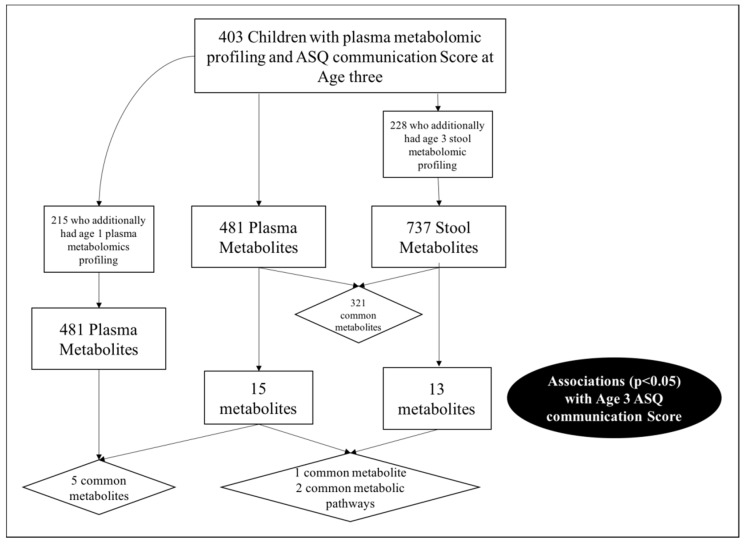
Study schematic and cross-over in communication score associated metabolites.

Both analyses identified metabolites of tryptophan metabolism and tyrosine metabolism as significantly dysregulated between children with an ASQ communication score indicating “on schedule” and those requiring “further monitoring/evaluation” (Figure 1).

**Figure 2:**
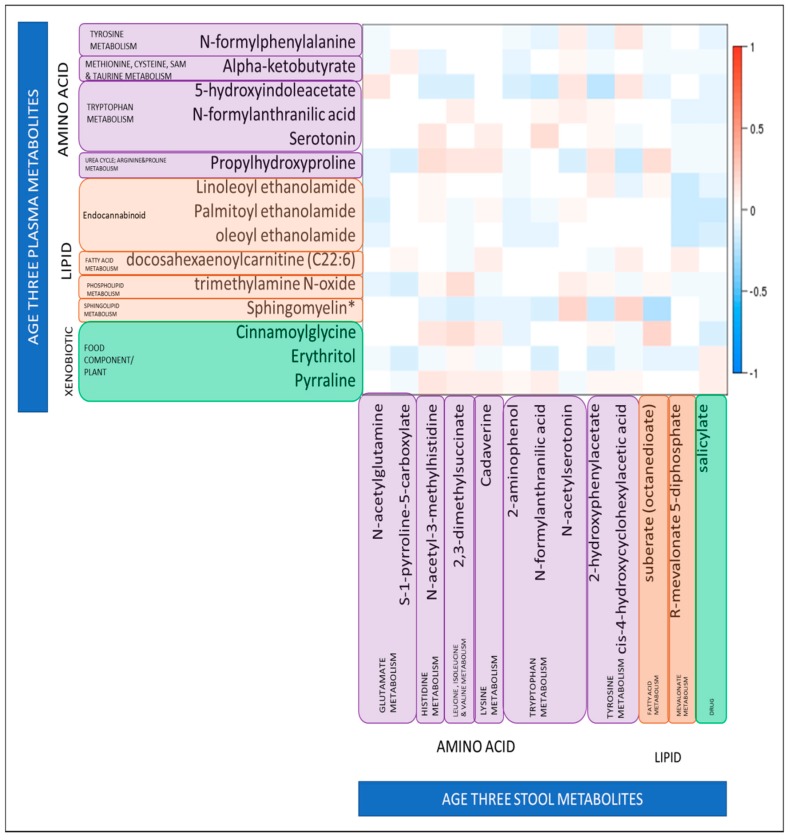
Relationship between levels of 15 plasma metabolites identified as significantly associated with binary ASQ score at age three, and 13 stool metabolites identified as significantly associated with binary ASQ score at age three. Relationships are quantified by Spearman’s Correlation Coefficients; color indicates direction of correlation (red is a positive correlation, blue is a negative correlation), darker shades indicate the significance of the association. White indicates no significant correlation. * Sphingomyelin (d18:1/25:0, d19:0/24:1, d20:1/23:0, d19:1/24:0).

Higher levels of N-formylanthranilic acid, a metabolite of tryptophan metabolism, were associated with a higher ASQ communication score in both plasma (odds ratio (OR): 0.05, 95% confidence interval (CI) 2.6 × 10^−3^, 0.75, *p* = 0.043) and stool (OR: 0.38, 95%CI 0.15, 0.93, *p* = 0.039). In order to explore this further, we looked at the correlation between plasma stool levels of N-formylanthranilic acid and stool levels of N-formylanthranilic acid among the children with measures of both at age three, to determine whether there was a relationship between levels of this metabolite in different biosamples. However we found that levels were not strongly correlated; Spearman *r* = 0.07, *p* = 0.3. We further sought to determine of there was any correlation between any of the 15 significant plasma metabolites with the 13 stool metabolites; but again we found little evidence of correlation (Figure 2). The strongest negative correlations were between plasma sphingomyelin and stool suberate (*r* = −0.26, *p* = 6.7 × 10^−5^) and between plasma 5-hydroxyindolacetate and stool 2-hydroxyphenylacetate (*r* = −0.24, *p* = 3.3 × 10^−4^). The strongest positive correlations were between plasma sphingomyelin and stool N-acetylserotonin (*r* = 0.24, *p* = 2.8 × 10^−4^) and between plasma cinnamoylglycine and stool suberate (*r* = 0.22, *p* = 6.9 × 10^−4^). However, it should be noted that although significant the correlation coefficients were not high for any of these plasma–stool metabolite pairs.

### 2.5. Autism

The VDAART children are now between 6 and 8.5 years old. Beginning at the year six VDAART follow up visit, we asked parents if their child “had ever received a physician diagnosis of autism”. To date, 18 children in VDAART have been diagnosed with autism according to parental report, including 9 (2.2%) of the 403 children with age three ASQ assessment and plasma metabolomic profiling. The rate of diagnosis among the children in the “requiring monitoring or further evaluation” category was 5/38 (13.2%) which was significantly higher (*p* = 5.5 × 10^−4^) than in the “on schedule” category; 4/365 (1.1%). This was also evident when considering the age two ASQ communication scores; with rates of 10.4% versus 0.9% (*p* = 0.001) in the “requiring monitoring or further evaluation” and the “on schedule” categories, respectively (Supplementary Table S3).

Accordingly, the binary ASQ score (Model 1) had moderate predictive ability for age 8 asthma in a receiver operator characteristic (ROC) curve analysis (area under the curve (AUC): 0.736 (95% CI: 0.563, 0.909)) (Supplementary Figure S2). Although this classifier had high sensitivity, (91.6%), its specificity was only 55.6% (Table 4). A summary score based on the 15 plasma significant metabolites (Model 2) demonstrated marginal, and non-significant, improvement in the AUC (0.759 (95% CI: 0.603, 0.915)) over the ASQ score model. Although specificity was higher (66.7%), sensitivity was lower (78.7%). However, when levels of all 15 metabolites (Model 3) were included the resulting model had a significantly higher AUC: 0.924 (95% CI: 0.867, 0.980), *p* = 0.034, than Model 1, and Model 3 demonstrated both high sensitivity (88.9%) and specificity (84.5%) for the prediction of autism by age 8.

## 3. Discussion

Impaired communication is a defining characteristic of autism spectrum disorders, which are now recognized as one of the most serious health problems in the world next to Acquired Immuno-Deficiency Syndrome (AIDS), cancer, and diabetes [14]. Despite their high prevalence and the public health burden they impart, there are still no tests that offer a reliable confirmation of a clinical diagnosis of ASD; that provide efficient screening of individuals presenting with behavioral features suggestive of ASD; or that aid in early detection of ASD [15]. Consequently, novel screening approaches are critical, particularly those that could be applied to young children. Metabolomics offers a particularly compelling avenue, given the demonstrated metabolic changes that accompany ASD, and the ability of metabolites and metabolomic profiles to act as biomarkers whilst also informing on underlying biological mechanisms.

In this study, we explored the hypothesis that metabolomic biomarkers could help to identify children with poor communication skills, as assessed by the ASQ. We hypothesized that children with a low ASQ communication score at age three were more likely to be diagnosed with autism in the following years. Indeed, more than 10% of the children in the lowest two categories of ASQ communication score at age two years, and more than thirteen percent at age three, went on to be diagnosed with autism, compared to ~ 1% of children who were categorized as “on schedule” at these ages. Furthermore, although we did not identify a global difference in the metabolomes of children “on schedule” versus those requiring “further monitoring/evaluation”, we did identify dysregulation of metabolism in both the plasma and stool of children with low ASQ communication scores in a number of metabolomic pathways that have previously been associated with autism and ASD. In particular tryptophan biosynthesis, tyrosine metabolism and endocannabinoid metabolism.

Tryptophan serves as a precursor for a wide range of bioactive compounds, including major neurotransmitters and neuromodulators, and it is involved in both protein synthesis and bacterial degradation [15]. These crucial functionalities of tryptophan and its associated pathways underlie much of the evidence linking dysregulated tryptophan biosynthesis and metabolism to ASD [15]. The two main pathways of tryptophan metabolism lead to the synthesis of kynurenine and of serotonin [16]. The majority of bioavailable tryptophan, which comes mainly from the diet, enters the kynurenine pathway leading to the production of kynurenic acid, kynuramines, picolinic acid, quinolinic acid, Nicotinamide adenine dinucleotide (NAD), and Adenosine triphosphate (ATP). These downstream products are involved in the regulation of the central nervous system and of the immune system [17], both of which have been shown to be dysregulated among individuals with autism. Less than 2% of the bioavailable tryptophan is metabolized into serotonin [16]. Serotonin is a neurotransmitter involved in multiple aspects of brain function that may influence ASD both dynamically and across development, ranging from the regulation of mood, appetite and social interactions to a critical role in neuronal morphology and circuitry [16]. This is important, as disordered organization of the fronto-temporal lobes is one of the most consistent neuroanatomical findings in ASD patients [18]. Furthermore, serotonin is implicated in regulation of the circadian rhythm, which is often shown to be disrupted in individuals with ASD [19], through its downstream product, melatonin.

It has been shown that in individuals with ASD, the tryptophan biosynthesis pathway in the brain is preferentially biased toward the production of xanthurenic acid and quinolinic acid along the kynurenine pathway, at the expense of the serotonin pathway [20], and consequently serotonin levels have been demonstrated to be lower in the brains of individuals with ASD [21]. However, the opposite is true outside the blood–brain barrier. In fact, elevated whole blood serotonin, or hyperserotonemia, was identified as the first biomarker of ASD more than fifty years ago, and is apparent in roughly 30% of affected individuals [10]. This is in agreement with our findings of higher levels of plasma serotonin, and lower levels of N-formylanthranilic acid, a downstream product of the kynurenine pathway, in the plasma of children with poor ASQ communication skills, relative to those with “normal” communication development. The remaining ~4% of tryptophan that is not metabolized down either the kynurenine or serotonin pathway undergoes bacterial degradation prior to gut absorption [20], which likely explains why we also saw altered levels of tryptophan metabolites in the stool. What is particularly compelling in the literature to date is that dysregulated tryptophan metabolism appears to be specific to ASD, as it has not been observed in other cognitively impaired individuals [22].

In this population both the plasma and the stool metabolome also indicated disruption of tyrosine metabolism across communication score categories. Tyrosine metabolism has previously been implicated in autism in a study of urinary metabolomics of Italian children [20]. Interestingly, one of the replicated risk genes for ASD, *MET,* encodes a receptor tyrosine kinase, MET, which modifies a large number of neurodevelopmental events [23]. Three endocannabinoids, which are arachidonic acid-derived compounds, were identified among the significant plasma metabolites, and all three endocannabinoids were also associated with age three ASQ communication score when measured in the age one plasma samples. The endocannabinoid system plays a crucial neuromodulatory role in the regulation of emotional responses, behavioral reactivity and social interaction, and is also implicated in a number of common phenotypic characteristics of ASD including seizures, anxiety and poor memory processing [24]. The endocannabinoid system has, therefore, been suggested as a therapeutic target for these disorders. Our current findings in which endocannabinoids were shown to already be disturbed in affected children at age one year, suggest such therapeutics may be of particular use for early intervention or possible prevention. Despite a large body of evidence from animal models linking endocannabinoids to ASD, this represents the first evidence for such an association in a human population.

This study, therefore, provides compelling evidence to link ASQ communication to dysregulated metabolic processes, which may indicate risk of ASD. Nevertheless, there were a number of limitations. One of the biggest was sample size, the prevalence of ASDs is approximately 1% in the US population aged <8 years [16]. Within this subset of the VDAART population, the prevalence of parent-reported autism diagnosis was 2%, which may reflect the higher percentage of males in the population, nevertheless, we were underpowered to explore ASD as an outcome in VDAART. For this reason, we chose to study the ASQ communication score as a proxy for autism risk based on the findings of Hardy et al. [6], and we demonstrated good power for this endpoint in our population. We noted differences in the prevalence of children in the different ASQ score categories between the three sites; specifically in San Diego, no children were in the lowest communication score category. The San Diego site also had the highest socioeconomic status of the three cohorts, and this discrepancy in score may reflect unmeasured biased relating to the parents of the children that we cannot control for. However, we did adjust for maternal education level and maternal marital status, and stratification of the plasma logistic regression models by site indicated that overall the site differences in ASQ scores were not influencing our results and conclusions. We additionally adjusted for important factors such as sex and race, as well as asthma status and vitamin D supplementation to account for the nature of the VDAART study. Finally, we were limited in our ability to study all the relevant metabolites in the pathways of interest, there are currently no profiling methods that provide coverage of the entire metabolome and consequently there may be important ASD related pathways that we were not able to analyze at all. Many of our associations were not robust to correction for multiple testing, however given the biological relevance of our significant metabolites this may purely be a reflection of the limitations discussed above.

We observed a significant association between ASQ communication score and autism diagnosis in this population, however this should be interpreted with caution due to the limited number of cases. Furthermore, although this classifier demonstrates impressive specificity, sensitivity was poor. This finding supports the argument that more objective and quantifiable markers are crucial. A summary score based on the significant plasma metabolites, moderately improved the AUC and the sensitivity of classification, but at the expense of specificity. In contrast, a classifier based on the relative measures of all 15 metabolites, significantly improved the AUC and maintained impressive sensitivity and specificity. Again, this should be interpreted with caution due to sample size, the likelihood of overfitting and the fact the metabolites were identified using the ASQ proxy. Furthermore, as our biomarker panel was comprised of 15 metabolites, further study will be necessary to identify a potential subset of these, or related metabolites that may be more parsimonious and practical in clinical settings. Nevertheless, these findings provide early evidence to suggest that the development of metabolite based biomarkers that can outperform existing measures of autism prediction and diagnosis may be feasible.

We do not have detailed information on the physician diagnosed cases, as autism was assessed by parental report of a physician diagnosis. It is also possible that there is observation bias in that all low ASQ communication score subjects were not specifically evaluated for autism. ASD, by definition, represents a heterogeneous spectrum of neurodevelopmental conditions rather than a single disease, and it is feasible they do not all share the same metabolomic profile. It has been noted that even among subjects with ASD, the level of tryptophan can vary considerably in patients with autistic disorder as compared to Asperger’s syndrome [15].

In the comparison of the plasma and stool metabolomes, it was of interest to note that while similar pathways were highlighted, in particular tryptophan and tyrosine metabolism, the constituent metabolites differed. The only exception was N-formylanthranilic acid, and here we could identify no direct correlation between the levels of this metabolite in the stool and the plasma samples. This may reflect the timing of sample collection; the stool samples were collected up to 48 h before the plasma. However, it is likely also a reflection of the different stages of metabolism represented by the stool versus the plasma metabolome. It is the ability of different bio samples to capture differing aspects of dysregulated metabolism that has led to the argument that integrating multiple bio samples may provide “better clues to biological and pathological pathways” underlying disease [25]. However, the statistical methods for doing so remain underdeveloped, and in particular there is little literature directly comparing stool versus plasma metabolites. Further work is required to better understand these relationships.

## 4. Materials and Methods

### 4.1. Study Population

This study was nested within the Vitamin D Antenatal Asthma Reduction Trial (VDAART); which aimed to assess the potential of vitamin D supplementation in pregnant women to prevent asthma in their offspring. The study has been described in detail previously [11]. Briefly, pregnant non-smoking women between 10 to 18 weeks of gestation who had a history of asthma, eczema, or allergic rhinitis, or who conceived the child with a man with a history of such diseases were recruited from three sites across the USA; Boston, San Diego and St Louis, between 2009 and 2011. Women were randomized 1:1 to a daily dose of 4000 International Units (IU) vitamin D_3_ or a placebo tablet until delivery. All women additionally received a daily multivitamin containing 400 IU vitamin D_3_. VDAART was approved by the Institutional Review Boards (IRB) of the participating Clinical Centers and the Data Coordinating Center, with pregnant women signing informed consent at the enrollment visit covering both primary and secondary analyses of data.

### 4.2. Metabolomic Profiling

All VDAART participants followed after birth (*n* = 806) were asked to provide blood and stool samples at age 3 years. Parents collected stool samples from either a dirty diaper or a “potty hat” within the 48 h before their year three visit using provided collection kits. They then stored them in a home freezer before delivery to the clinical center in a freezer pack. Stool was not collected if the infant had used antibiotics in the past 7 days. Fecal samples were stored at the clinical centers at −80 °C and then delivered to Metabolon (Durham, NC, USA) where sample preparation and analysis was performed. Samples (~50 mg) were homogenized in methanol at 50 mg/mL for metabolite extraction. The supernatant was separated from debris and precipitates by centrifugation, divided into five aliquots for four different analysis conditions plus one backup sample, and placed into a TurboVap (Zymark, Hopkinton, MA, USA) for solvent removal. Dried samples were stored under nitrogen gas overnight until analysis.

Blood was collected at the year one and year three in-person visit then shipped to the Data Coordinating Center in Boston, where processing and aliquoting was done. Plasma was separated and immediately stored at −80 °C until global metabolomic profiling at Metabolon.

Sample profiling for both stool and plasma was performed using ultrahigh performance liquid chromatography-tandem mass spectroscopy (UPLC-MS/MS) and four platforms covering a broad range of the metabolome: (1) the first aliquot was analyzed using positive ion mode, chromatographically optimized for more hydrophilic compounds. The extract was gradient eluted from a C18 column (Waters UPLC BEH C18 2.1 × 100 mm, 1.7 µm) using water and methanol, containing 0.05% perfluoropentanoic acid (PFPA) and 0.1% formic acid (FA); (2) the second aliquot was also analyzed using positive ion mode, however it was chromatographically optimized for more hydrophobic compounds. The extract was gradient eluted from the same C18 column as above using methanol, acetonitrile, water, 0.05% PFPA and 0.01% FA operated at an overall higher organic content; (3) The third aliquot was analyzed using negative ion mode with a separate dedicated C18 column. The basic extracts were gradient eluted from the column using methanol and water with 6.5mM ammonium bicarbonate at pH 8; (4) The fourth aliquot was analyzed via negative ionization following elution from a Hydrophilic Interaction Chromatography (HILIC) column (Waters UPLC BEH Amide 2.1 × 150 mm, 1.7 µm) using a gradient consisting of water and acetonitrile with 10mM ammonium formate, pH 10.8.

Metabolites were analyzed as measured LC-MS peak areas and identified by their mass-to-charge ratio (*m/z*), retention time (rt), and through a comparison to library entries of purified known standards. The blood samples were processed in two batches sent six months apart including both age one and age three (batch one *n* = 245; batch two *n* = 688) then merged and scaled together based on equivalence of the control groups. If a metabolite was missing in 50% or more of the samples from either dataset, it was excluded from further analysis. In the stool samples, which were all processed in the same batch, intestinal metabolite relative abundances were normalized to sample mass (mg) [26]. Metabolites with a signal-to-noise ratio <10 were considered unquantifiable and excluded, as were metabolites with undetectable/missing levels for >10% of the samples. All remaining missing values were imputed with half the minimum peak intensity for that metabolite across the whole population, then data were pareto scaled to account for the differences in the scales of measurements across the metabolome. In both datasets metabolites were log-transformed to create approximately Gaussian distributions and to stabilize variance. Principal components analysis (PCA) of metabolite relative abundances was performed to look for outliers and age trends. One stool sample was over 10 standard deviations from the mean on principal component (PC) 1 and over 6 standard deviations from the mean on PC2 and was therefore excluded from analysis.

### 4.3. Ages and Stages Questionnaire (ASQ) Assessment

The Ages and Stages Questionnaire, 3rd Edition (ASQ-3) (https://agesandstages.com/; Paul H. Brookes Publishing Co., Inc.) [5] was administered to primary caregivers of the VDAART offspring at ages one, two and three years in person at the annual visit. The ASQ-3 is a validated and widely used tool that assesses five developmental domains: gross motor skills, fine motor skills, problem solving ability, personal/social skills and communication, with six questions in each category resulting in a domain specific score. Standardized domain specific scores are then compared to the expected mean score obtained from a reference distribution of scores within age-groups, and categorized as: (i) “On Schedule for developing normally” (above the mean); (ii) “Requires Monitoring” (1–2 standard deviations from the mean); (iii) “Needs further evaluation” (>2 standard deviations from the mean). For these analyses we focused on communication skill using a binary cut off comparing those “On schedule for developing normally” versus those either requiring “further evaluation” or “monitoring”, based on the recommendations of Hardy et al. [6].

### 4.4. Statistical Analysis

#### 4.4.1. Plasma and Stool Metabolome

In order to determine the ability of the overall metabolome to discriminate children who, according to ASQ communication score, require further monitoring or evaluation from those on schedule for developing normally, we conducted partial least squares discriminant analysis (PLS-DA) using both the (i) plasma metabolome and (ii) the stool metabolome. We computed the R^2^ and Q^2^ values for the first component, and ran a permutation test to assess the robustness of the model. We also identified those metabolites with the greatest Variable Importance in the Projection (VIP) score for the first component. The VIP score provides an estimate of the importance of each variable in the projection of the data onto the plane of greatest separation. We used a conservative cut off of VIP score > 2 to identify important discriminatory metabolites. We then sought to identify individual plasma and stool metabolites that may be associated with communication score using multivariable logistic regression models adjusting for child’s race, child’s sex, maternal education, maternal marital status, vitamin D treatment group and clinical site. For those metabolites that were significant at age three in plasma, we also determined the association of these metabolites as measured in the year one samples with age three ASQ score. Finally, we assessed the overlap and correlation between the metabolites that were significant in the stool, and those that were significant in the year three plasma. Using the powerLogisticCon function from the R package ‘powerMediation’, we determined our power to detect a significant association between binary ASQ score and a metabolite assuming an alpha of 0.05, an event rate of 0.09 for plasma (38/403) and of 0.11 for stool (24/228) across a range of odds ratios (Supplementary Table S8). For the plasma analysis we had a power of 85% to detect an OR < 0.6 and 93% power to detect an OR > 1.8. Similarly, for stool we had a power of 99% for an OR < 0.4, and 89% for an OR > 2.

#### 4.4.2. Correction for Multiple Testing

There are currently no consensus standards for multiple testing correction in metabolomics; methods applied to other ‘omic’ datatypes such as the Bonferroni correction, and even more liberal corrections are considered too stringent for metabolomics data due to the high correlation of metabolites that are closely linked together through biological pathways. Therefore, we report a nominal *p*-value significance of *p* < 0.05 throughout. However, we also explore an additional multiple-testing correction which partially takes into account the presence of highly correlated metabolites mapping to the same biological pathway, using a principal components analysis (PCA) approach to identify the number of effective tests [12,13]. We applied PCA to the metabolites that passed quality control (QC) and processing and determined the number of components required to explain 50% of the variance in the data (i.e., the number of effective tests). The adjusted *p*-value threshold was then calculated as α/m where α denotes the nominal *p*-value threshold of 0.05, and m denotes the number of effective (i.e., independent) tests. This was applied to both the plasma and the stool metabolites separately.

#### 4.4.3. Diagnosing and Predicting Autism

We compared the rates of autism diagnosis between the communication score categories to determine whether children “requiring further monitoring/evaluation” at ages 2 and 3 were more likely to go on to develop autism by age eight than those “on schedule for developing normally”. We used the Fishers exact test to account for small cell counts. Next, we aimed to determine whether the plasma metabolites identified as being associated with ASQ at age three had any predictive ability for autism by age eight. We compared three different predictive models using ROC cures and the corresponding AUC; Model 1: binary ASQ score at age three; Model 2: a summary score, generated by taking the first five principal components of the 15 plasma metabolites that were significantly associated with communication score at age three. Five PCs were chosen, as this was the number required to explain 50% of the variance in the data; and Model 3: Levels of all 15 significant metabolites. For the Models 2 and 3, the sensitivity and specificity were computed based on the optimal cut-off to maximize sensitivity and specificity weighting both equally, as determined using the ‘ROCR’ package in R [27].

All analyses were conducted in R version 3.5.0 and all statistical tests were two-sided.

## 5. Conclusions

Despite the relatively limited sample size, this study still represents one of the largest to consider metabolomics and neurodevelopment. It is unique in the inclusion of both blood and stool metabolites and is, to our knowledge, the first to explore ASQ scores and metabolomics. We identified a number of metabolomic pathways and metabolites with biologically plausible relationships with impaired development of communication skills and with autism risk. Finally, we demonstrated the predictive ability of these metabolites, providing evidence that ASQ communication score and metabolomic profiling may provide alternative approaches for early diagnosis of ASDs.

## Figures and Tables

**Table 1:** Baseline characteristics of 403 children from the Vitamin D Antenatal Asthma Reduction Trial (VDAART) with plasma metabolomic profiling and an Ages and Stages Questionnaire (ASQ) score at age 3.

Baseline Characteristic		Age 3ASQ Communication Score						*p*-Value
		On Schedule (*n* = 365)		Needs Monitoring (*n* = 23)		Requires Further Evaluation (*n* = 15)		
		*n*	%	*n*	%	*n*	%	
Sex	Female	176	48.2%	9	39.1%	3	20.0%	0.076
	Males	189	51.8%	14	60.9%	12	80.0%	
Site	San Diego	131	35.9%	6	26.1%	0	0.0%	0.002
	Boston	72	19.7%	5	21.7%	9	60.0%	
	St Louis	162	44.4%	12	52.2%	6	40.0%	
Race	Black	173	47.4%	13	56.5%	9	60.0%	0.725
	White	122	33.4%	6	26.1%	5	33.3%	
	Other	70	19.2%	4	17.4%	1	6.7%	
Treatment	Vitamin D	186	51.0%	12	52.2%	7	46.7%	0.940
	Placebo	179	49.0%	11	47.8%	8	53.3%	
Asthma/Wheeze	Yes	93	25.5%	5	21.7%	6	40.0%	0.414
	No	272	74.5%	18	78.3%	9	60.0%	
Maternal Marital Status	Married	179	49.0%	9	39.1%	4	26.7%	0.449
	Not married/not living together	88	24.1%	8	34.8%	6	40.0%	
	Not married - living together	86	23.6%	6	26.1%	5	33.3%	
	Separated/Divorced	12	3.3%	0	0.0%	0	0.0%	
Maternal Educational Level	Less than high school	45	12.3%	3	13.0%	3	20.0%	0.057
	High school, Technical school	96	26.3%	10	43.5%	5	33.3%	
	Some college	84	23.0%	5	21.7%	6	40.0%	
	College graduate/Graduate school	140	38.4%	5	21.7%	1	6.7%	
Body Mass Index (BMI) at age 3	Mean (standard deviation, SD)	16.7 (1.9)		16.4 (1.3)		17.2 (2.3)		0.528
Age 1 Communication Score	On Schedule	308	84.4%	18	78.3%	14	93.3%	0.200
	Needs monitoring	5	1.4%	1	4.3%	1	6.7%	
	Requires further evaluation	2	0.5%	0	0.0%	0	0.0%	
	Missing	50	13.7%	4	174%	0	0.0%	
Age 2 Communication Score	On Schedule	315	86.3%	11	47.8%	5	33.3%	8.0 × 10^−9^
	Needs monitoring	24	6.6%	5	21.7%	5	33.3%	
	Requires further evaluation	7	1.9%	3	13.0%	4	26.7%	
	Missing	19	5.2%	4	17.4%	1	6.7%	
Stool samples available	Yes	204	55.9%	14	60.9%	10	66.7%	-

**Table 2:** Plasma metabolites associated (*p* < 0.05) with binary ASQ communication score category.

Metabolite	Super Pathway	Sub Pathway	HMDB ID ^a^	OR (95% Confidence Interval, CI)	*p*-Value
N-formylphenylalanine *	Amino Acid	Tyrosine Metabolism	-	4.1 × 10^−3^ (4.1 × 10^−2^,0.11)	0.002
trimethylamine N-oxide *	Lipid	Phospholipid Metabolism	HMDB00925	30.13 (2.74,337.23)	0.005
cinnamoylglycine *	Xenobiotics	Food Component/Plant	HMDB11621	4.41 (1.52,12.80)	0.006
linoleoyl ethanolamide *	Lipid	Endocannabinoid	HMDB12252	12.38 (1.67,92.73)	0.013
palmitoyl ethanolamide	Lipid	Endocannabinoid	HMDB02100	141.67 (2.25,9324.32)	0.019
5-hydroxyindoleacetate *	Amino Acid	Tryptophan Metabolism	HMDB00763	0.08 (0.01,0.64)	0.023
erythritol	Xenobiotics	Food Component/Plant	HMDB02994	21.39 (1.31,300.33)	0.024
pyrraline *	Xenobiotics	Food Component/Plant	HMDB33143	0.11 (0.01,0.68)	0.024
sphingomyelin (d18:1/25:0, d19:0/24:1, d20:1/23:0, d19:1/24:0) *	Lipid	Sphingolipid Metabolism	-	0.02 (3.3 × 10^−4^,0.62)	0.034
docosahexaenoylcarnitine (C22:6)	Lipid	Fatty Acid Metabolism (Acyl Carnitine)	-	6.28 (1.07,34.75)	0.037
prolylhydroxyproline	Amino Acid	Urea cycle; Arginine and Proline Metabolism	HMDB06695	95.30 (1.29,7378.98)	0.038
alpha-ketobutyrate	Amino Acid	Methionine, Cysteine, SAM and Taurine Metabolism	HMDB00005	3.98 (1.04,14.72)	0.040
N-formylanthranilic acid *	Amino Acid	Tryptophan Metabolism	HMDB04089	0.05 (3.3 × 10^−4^,0.75)	0.043
Serotonin *	Amino Acid	Tryptophan Metabolism	HMDB00259	5.75 (1.03,32.63)	0.046
oleoyl ethanolamide	Lipid	Endocannabinoid	HMDB02088	12.75 (0.99,161.42)	0.048

* Significant in the 3 category communication score analysis. Human Metabolome Database Identifier.

**Table 3:** Stool metabolites associated with binary ASQ communication score category.

Metabolite	Super Pathway	Sub Pathway	HMDB ID	OR (95% CI)	*p*-Value
salicylate *	Xenobiotics	Drug	HMDB01895	0.3 (0.13,0.64)	0.003
R-mevalonate 5-diphosphate *	Lipid	Mevalonate Metabolism	HMDB01981	0.38 (0.18,0.69)	0.004
N-acetylglutamine *	Amino Acid	Glutamate Metabolism	HMDB06029	0.25 (0.09,0.65)	0.005
2-hydroxyphenylacetate	Amino Acid	Tyrosine Metabolism	HMDB00669	0.44 (0.22,0.83)	0.014
suberate (octanedioate) *	Lipid	Fatty Acid, Dicarboxylate	HMDB00893	0.31 (0.12,0.78)	0.015
2-aminophenol	Amino Acid	Tryptophan Metabolism	-	0.43 (0.21,0.85)	0.016
cadaverine *	Amino Acid	Lysine Metabolism	HMDB02322	0.55 (0.33,0.89)	0.017
N-acetyl-3-methylhistidine	Amino Acid	Histidine Metabolism	-	0.57 (0.34,0.96)	0.034
N-formylanthranilic acid	Amino Acid	Tryptophan Metabolism	HMDB04089	0.38 (0.15,0.93)	0.039
cis-4-hydroxycyclohexylacetic acid	Amino Acid	Tyrosine Metabolism	HMDB00451	0.54 (0.29,1.00)	0.043
2,3-dimethylsuccinate	Amino Acid	Leucine, Isoleucine and Valine Metabolism	-	0.55 (0.31,1.00)	0.044
S-1-pyrroline-5-carboxylate	Amino Acid	Glutamate Metabolism	HMDB01301	0.55 (0.30,1.00)	0.048
N-acetylserotonin	Amino Acid	Tryptophan Metabolism	HMDB01238	0.50 (0.24,0.97)	0.050

* Significant in the 3 category communication score analysis.

**Table 4:** Comparison of three different models for the prediction of autism by age 8.

Classifier.	Area under the Curve (AUC) (95% CI)	Performance Compared to Model 1	Sensitivity	Specificity
Model 1: Binary ASQ Communication Score	0.736 (0.563, 0.909)	-	55.6%	91.6%
Model 2: Metabolite Summary Score	0.759 (0.603, 0.915)	*p = 0.635*	66.7%	78.7%
Model 3: Metabolite Levels	0.924 (0.867, 0.980)	*p = 0.034*	88.9%	84.5%

## Supplementary Materials

The following are available online at https://www.mdpi.com/2218-1989/9/3/42/s1: Supplementary Figure S1: Trajectory of Communication Score Category at ages 1,2 and 3 in 403 children with plasma metabolomics profiling; Supplementary Figure S2: (A) Scores plot for the PLS-DA Model Based on all 481 Plasma Metabolites Comparing Children with ASQ Assessed Communication Skills on Schedule for Developing Normally (*n* = 365) versus those Requiring Further Monitoring/Follow up (n-38); R^2^ = 0.13; Q^2^ = −0.21; permutation *p*-value = 0.994; (B) Plasma Metabolites with a VIP score >2, Indicating the Greatest Ability to Discriminate Between the Two Groups; Supplementary Figure S3: (A) Scores plot for the PLS-DA Model Based on all 737 Stool Metabolites Comparing Children with ASQ Assessed Communication Skills on Schedule for Developing Normally (*n* = 204) versus those Requiring Further Monitoring/Follow up (*n* = 24); R^2^ = 0.10; Q^2^ = −1.13; permutation *p*-value = 0.985; (B) Plasma Metabolites with a VIP score >2, Indicating the Greatest Ability to Discriminate Between the Two Groups; Supplementary Figure S4: Receiver Operator Characteristic Curves and Corresponding AUCS for the prediction of autism by age 8 according to three models; Table S1: Baseline characteristics of 228 children from VDAART with plasma and stool metabolomic profiling and an ASQ score at age 3; Table S2: Correlation between Communication Score and Scores in the three other ASQ domains; Table S3: 481 Plasma Metabolites that Passed QC with information on Profiling Platform, Superpathway, Subpathway and HMDB ID; Table S4: Association Between 15 Significant Plasma Metabolites and Binary ASQ score, Stratified by Study Site; Table S5: 737 Stool Metabolites that Passed QC with information on Profiling Platform, Superpathway, Subpathway and HMDB ID; Table S6: Metabolites Associated with Binary ASQ Communication Score at age three in blood plasma samples from ages 1 and 3; Table S7: Subsequent Diagnoses of Autism by age eight stratified by ASQ communication score category at age 2 and at age 3 among 403 children with ASQ communication score and metabolomics profiling; Table S8: Power Analysis for the Plasma and Stool Logistic Regression Models; assuming an alpha of 0.05 and an event rate of 0.09 and a sample size of 403 for plasma and an event rate of 0.11 and a sample size of 228 for stool
