# Supplementary material for: Metabolomics and Communication Skills Development in Children; Evidence from the Ages and Stages Questionnaire
Source: Metabolites. 2019 Mar 5;9(3):42. doi: 10.3390/metabo9030042 (PMC6468693; doi:10.3390/metabo9030042)
Supplement: Supplementary file 1 [file metabolites-09-00042-s001.zip › Supplementary Material.pdf]

Supplementary Figures

Supplementary Figure S1: Trajectory of Communication Score Category at ages 1, 2 and 3 in 403 Children with plasma metabolomics profiling

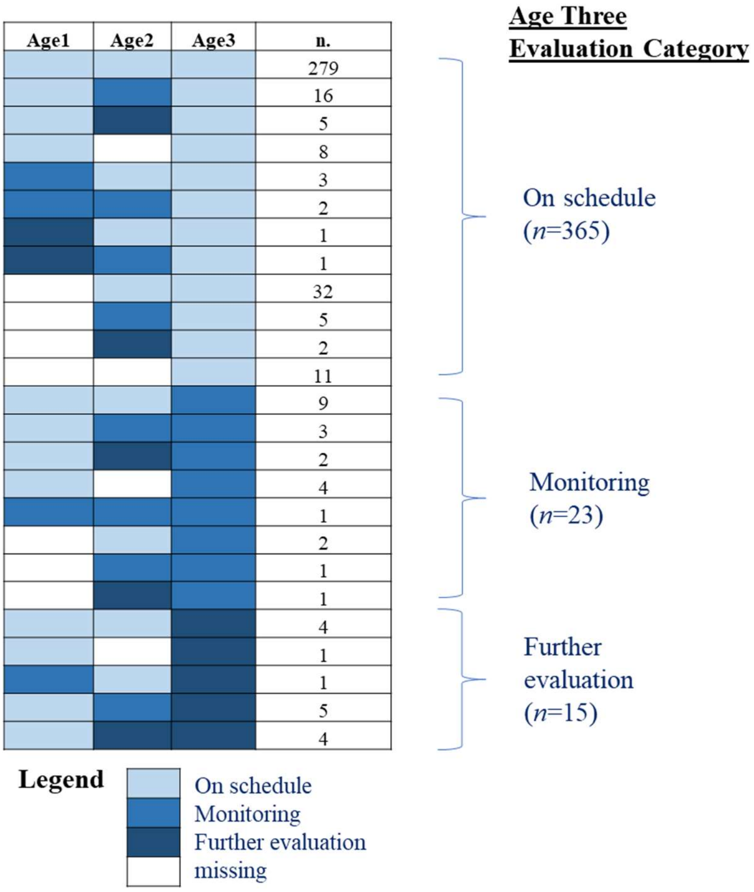

Supplementary Figure S2: (A) Scores plot for the PLS-DA Model Based on all 481 Plasma Metabolites Comparing Children with ASQ Assessed Communication Skills on Schedule for Developing Normally (n=365) versus those Requiring Further Monitoring/Follow up (n-38); R^2=0.13; Q^2=-0.21; permutation p-value = 0.994; (B) Plasma Metabolites with a VIP score >2, Indicating the Greatest Ability to Discriminate Between the Two Groups

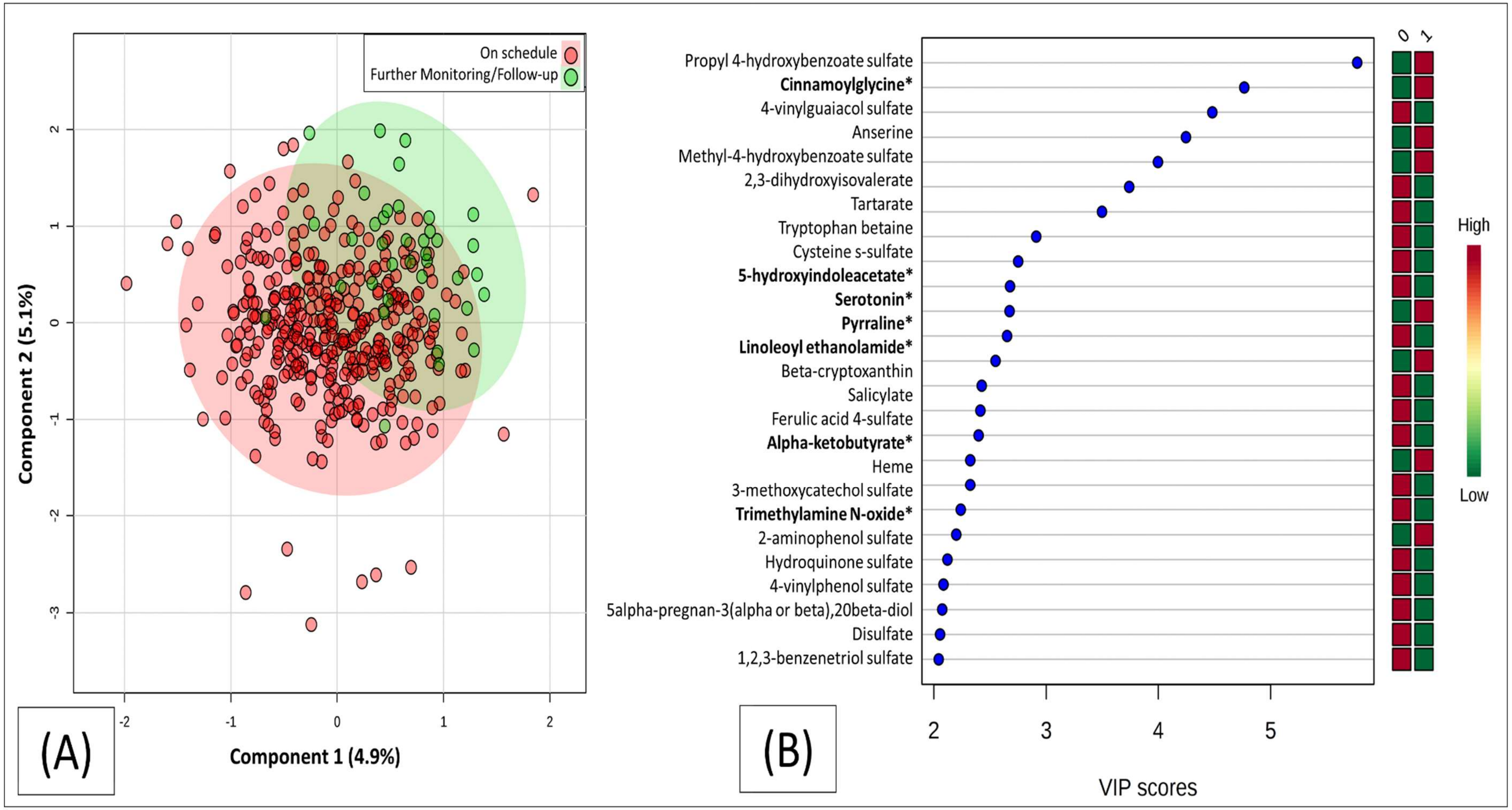

****Metabolites that were significantly associated with binary communication score (p<0.05) in the logistic regression models***

Supplementary Figure S3: (A) Scores plot for the PLS-DA Model Based on all 737 Stool Metabolites Comparing Children with ASQ Assessed Communication Skills on Schedule for Developing Normally (n=204) versus those Requiring Further Monitoring/Follow up (n=24); R^2=0.10; Q^2=-1.13; permutation p-value = 0.985; (B) Plasma Metabolites with a VIP score >2, Indicating the Greatest Ability to Discriminate Between the Two Groups

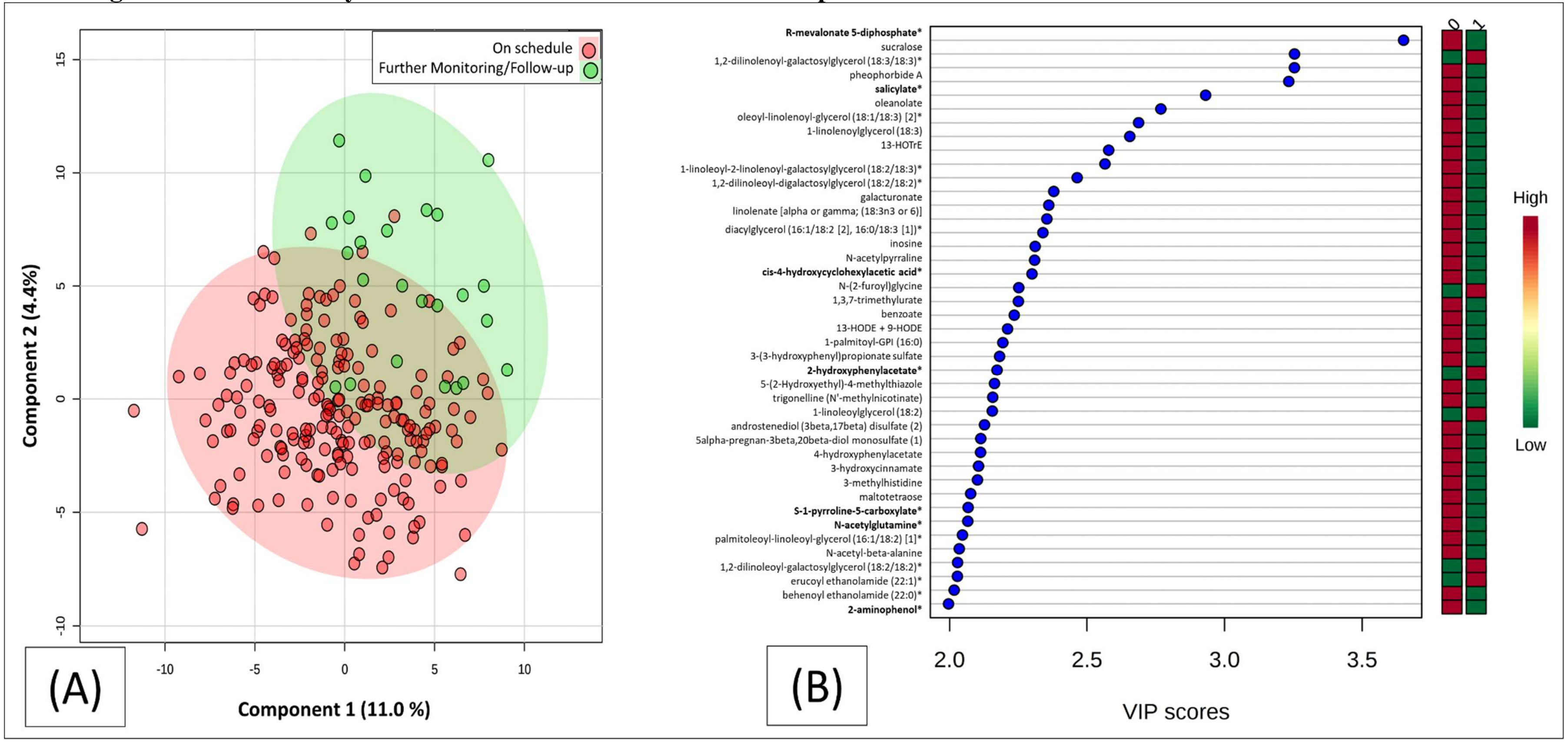

**Metabolites that were significantly associated with binary communication score (p<0.05) in the logistic regression model*

Supplementary Figure S4: Receiver Operator Characteristic Curves and Corresponding AUCS for the prediction of autism by age 8 according to three models

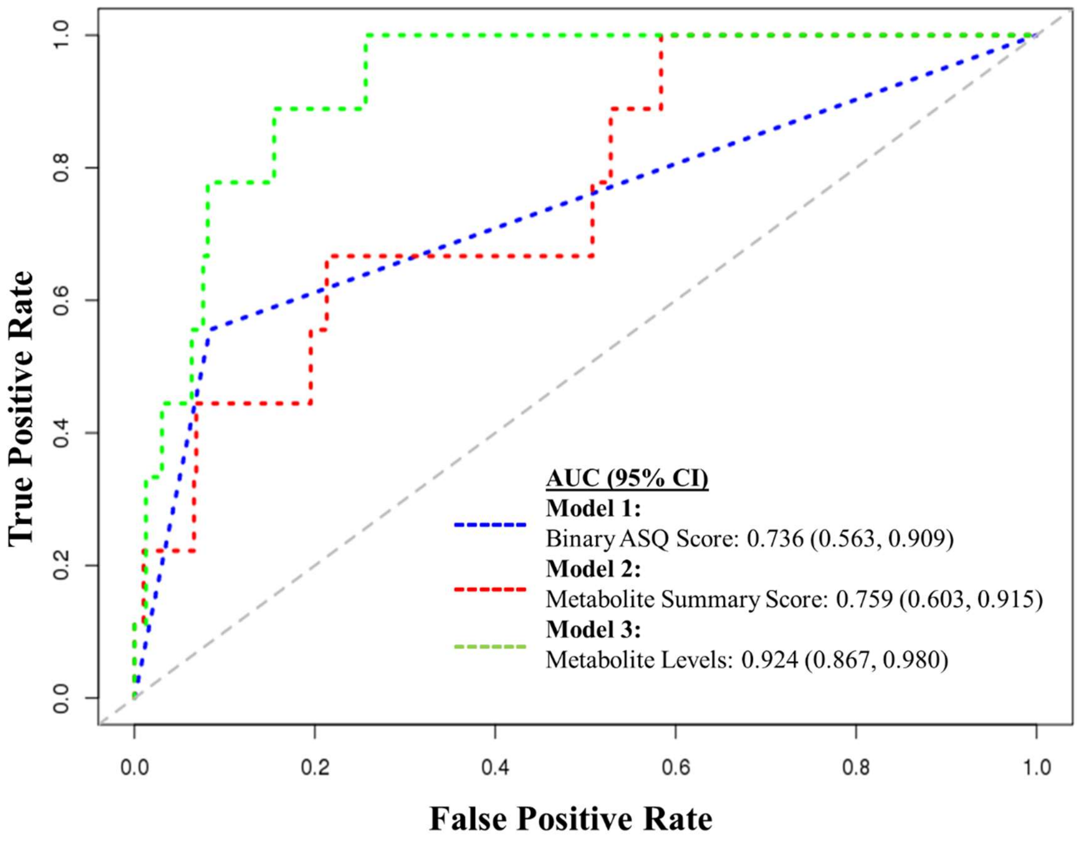

AUC- Area under the curve

Supplementary Tables

Table S1: Baseline characteristics of 228 children from VDAART with plasma and stool metabolomic profiling and an ASQ score at age 3

		On Schedule (n=204)		Needs monitoring (n=14)		Requires further evaluation (n=10)		
		n	%	n	%	n	%	p-value
Sex	Female	99	48.5%	5	35.7%	3	30.0%	0.356
	Males	105	51.5%	9	64.3%	7	70.0%	
Site	San Diego	64	31.4%	4	28.6%	0	0.0%	0.108
	Boston	31	15.2%	2	14.3%	4	40.0%	
	St Louis	109	53.4%	8	57.1%	6	60.0%	
Race	Black	89	43.6%	8	57.1%	7	70.0%	0.467
	White	42	20.6%	1	7.1%	1	10.0%	
	Other	73	35.8%	5	35.7%	2	20.0%	
Treatment	Vitamin D	102	50.0%	7	50.0%	5	50.0%	1.00
	Placebo	102	50.0%	7	50.0%	5	50.0%	
Asthma/Wheeze	Yes	80	39.2%	5	35.7%	6	60.0%	0.447
	No	124	60.8%	9	64.3%	4	40.0%	
Maternal Marital Status	Married	99	48.5%	6	42.9%	2	20.0%	0.530
	Not married/not living together	54	26.5%	5	35.7%	5	50.0%	
	Not married - living together	45	22.1%	3	21.4%	3	30.0%	
	Separated/Divorced	6	2.9%	0	0.0%	0	0.0%	
Maternal Educational Level	Less than high school	28	13.7%	2	14.3%	2	20.0%	0.119
	High school, Technical school	60	29.4%	5	35.7%	4	40.0%	
	Some college	40	19.6%	4	28.6%	4	40.0%	
	College graduate/Graduate school	76	37.3%	3	21.4%	0	0.0%	
BMI	mean (SD)	17.5 (2.3)		16.4 (1.1)		16.8 (2.0)		0.557
Age 1 Communication Score	On Schedule	173	84.8%	13	92.9%	9	90.0%	0.431
	Needs monitoring	3	1.5%	0	0.0%	1	10.0%	
	Requires further evaluation	1	0.5%	0	0.0%	0	0.0%	
	Missing	22	10.8%	1	7.1%	0	0.0%	
Age 2 Communication Score	On Schedule	181	88.7%	8	57.1%	5	50.0%	2.3×10^{-4}
	Needs monitoring	13	6.4%	2	14.3%	2	20.0%	
	Requires further evaluation	3	1.5%	1	7.1%	2	20.0%	
	Missing	7	3.4%	3	21.4%	1	10.0%	

Table S2: Correlation between Communication Score and Scores in the three other ASQ domains

	**Spearman *r* (p-value)**
**Fine Motor Skills Score**	0.40 ($<2.2\text{x}10^{-16}$)
**Gross Motor Skills**	0.30 ($6.8\text{x}10^{-10}$)
**Personal Social Skills**	0.45 ($<2.2.\text{x}10^{-16}$)
**Problem Solving Skills**	0.46 ($<2.2\text{x}10^{-16}$)

Table S3: 481 Plasma Metabolites that Passed QC with information on Profiling Platform, Superpathway, Subpathway and HMDB ID

See excel file

Table S4: Association Between 15 Significant Plasma Metabolites and Binary ASQ score, Stratified by Study Site

	San Diego		Boston		St Louis	
**Metabolite**	**OR (95% CI)**	**p-value**	**OR (95% CI)**	**p-value**	**OR (95% CI)**	**p-value**
**N-formylphenylalanine**	0.02(6.4x10^{-6},40.67)	0.309	3x10-4(6.8x10^{-7},0.07)	0.005*	0.01(9.9x10^{-5},1.02)	0.06
**Trimethylamine N-oxide**	15.2(0.07,2031.3)	0.288	247.4(4.44,27655.2)	0.011*	7.39(0.2,226.6)	0.258
**Cinnamoylglycine**	0.4(0.01,6.75)	0.58	4.35(0.9,21.71)	0.066	4.63(1.08,19.89)	0.037*
**Linoleoyl ethanolamide**	43.7(0.23,5154.9)	0.123	10.57(0.34,359.5)	0.178	7.29(0.56,84.4)	0.111
**Palmitoyl ethanolamide**	13082.1(0.25,6.4x10^{8})	0.078	19.6(0.01,36700.5)	0.441	65.2(0.46,7726.9)	0.085
**5-hydroxyindoleacetate**	0.04(3.5x10^{-4},1.95)	0.144	0.09(3.9x10^{-3},1.29)	0.098	0.31(4.0x10^{-3},11.47)	0.562
**Erythritol**	1261.4(1.73,1.9x10^{6})	0.036*	2.12(0.01,351.7)	0.776	9.81(0.26,297.9)	0.171
**Pyrraline**	0.05(3.7x10^{-4},2.4)	0.183	0.29(0.02,3.53)	0.367	0.07(2.6x10^{-3},1.31)	0.097
**Sphingomyelin (d18:1/25:0, d19:0/24:1, d20:1/23:0, d19:1/24:0)***	1.7x10-3(0.02,3.39)	0.142	0.01(9.2x10^{-6},3.36)	0.142	0.08(5.0x10^{-4},7.45)	0.305
**Docosahexaenoylcarnitine (C22:6)***	43.35(1.2,1655.5)	0.034*	5.81(0.18,156.0)	0.297	3.45(0.25,34.49)	0.315
**Prolylhydroxyproline**	385.68(0.03,1.9x10^{6})	0.159	0.07(1.8x10$^{-5,}$282.6)	0.529	751(3.14,2.3x10^{5})	0.019*
**Alpha-ketobutyrate**	3.53(0.16,49.44)	0.372	2.95(0.3,24.98)	0.324	4.12(0.63,23.48)	0.118
**N-formylanthranilic acid**	5.82(0.01,1771.8)	0.575	0.05(3.5x10^{-4},2.08)	0.202	0.03(3.0x10^{-4},1.25)	0.083
**Serotonin**	24.5(0.23,2320.6)	0.162	1.14(0.05,18.44)	0.928	15.49(1.5,178.4)	0.023*
**Oleoyl ethanolamide**	91.56(0.11,44714)	0.157	4.43(0.06,250.9)	0.478	12.74(0.44,334.5)	0.122

Logistic regression models were unadjusted due to convergence issues with small numbers
**Significant at the 95% confidence interval*

Table S5: 737 Stool Metabolites that Passed QC with information on Profiling Platform, Superpathway, Subpathway and HMDB ID

See excel file

Table S6: Metabolites Associated with Binary ASQ Communication Score at age three in blood plasma samples from ages 1 and 3

Metabolite	Super Pathway	Sub Pathway	HMDB ID	Age 1 Blood samples		Age 3 Blood samples		Correlation between Age 1 and Age 3 Levels*
				**OR (95% CI)**	**p-value**	**OR (95% CI)**	**p-value**	***r* (p-value)**
linoleoyl ethanolamide	Lipid	Endocannabinoid	HMDB12252	673.0 (13.3,4.8x10^4)	0.002	12.4 (1.7,92.7)	0.013	*0.18* (0.007)
oleoyl ethanolamide	Lipid	Endocannabinoid	HMDB02088	1320.1 (11.2.3x10^5)	0.004	12.7 (1,161.4)	0.048	*0.23* (0.001)
palmitoyl ethanolamide	Lipid	Endocannabinoid	HMDB02100	17911.5 (24.7,2.8x10^7)	0.007	141.7 (2.3,9324.3)	0.019	*0.26* (1.2x10^{-4})
docosahexaenoylcarnitine (C22:6)	Lipid	Fatty Acid Metabolism (Acyl Carnitine)	-	6.6 (1.1,40.6)	0.036	6.3 (1.1,34.8)	0.037	*0.13* (0.064)
sphingomyelin (d18:1/25:0, d19:0/24:1, d20:1/23:0, d19:1/24:0)	Lipid	Sphingolipid Metabolism	-	0.02 (3.3x10^{-8},0.4)	0.043	0.02 (5.7x10^{-4},0.62)	0.034	*0.37* (2.4x10^{-8})

**Spearman's correlation coefficient among 215 children with plasma metabolomics profiling at age 1 and age 3*

Table S7: Subsequent Diagnoses of Autism by age eight stratified by ASQ communication score category at age 2 and at age 3 among 403 children with ASQ communication score and metabolomics profiling

		Diagnosis of Autism by Age 8			
		Yes	No	*fishers p-value*	OR (95% CI), *p-value*
Age 2 Communication Score	On Schedule (n=331)	3 (0.9%)	328 (99.1%)	*0.001*	12.7 (3.0, 63.8); *$6.8x10^{-4}$*
	Needs monitoring/ Further Evaluation (n=48)	5 (10.4%)	43 (89.6%)		
Age 3 Communication Score	On Schedule (n=365)	4 (1.1%)	361 (98.9%)	*$5.5x10^{-4}$*	13.7 (3.4, 57.6); *$1.7x10^{-4}$*
	Needs monitoring/ Further Evaluation (n=38)	5 (13.2%)	33 (86.8%)		

Odds ratios are unadjusted due to small cell counts

Table S8: Power Analysis for the Plasma and Stool Logistic Regression Models; assuming an alpha of 0.05 and an event rate of 0.09 and a sample size of 403 for plasma and an event rate of 0.11 and a sample size of 228 for stool

Odds Ratio	Power	
	Plasma	Stool
0.2	100.0%	100.0%
0.4	100.0%	98.9%
0.6	84.9%	65.7%
0.8	25.7%	17.7%
1.2	18.6%	13.2%
1.4	50.4%	34.4%
1.6	78.6%	58.5%
1.8	93.1%	77.7%
2.0	98.2%	89.4%
2.2	99.6%	95.4%
2.4	99.9%	98.2%
2.6	100.0%	99.3%
2.8	100.0%	99.7%
3.0	100.0%	99.9%
3.2	100.0%	100.0%
3.4	100.0%	100.0%
3.6	100.0%	100.0%
3.8	100.0%	100.0%
4.0	100.0%	100.0%
